# Effect of Ginger on Relieving Nausea and Vomiting in Pregnancy: A Randomized, Placebo-Controlled Trial

**DOI:** 10.17795/nmsjournal11841

**Published:** 2014-04-17

**Authors:** Farzaneh Saberi, Zohreh Sadat, Masoumeh Abedzadeh-Kalahroudi, Mahboobeh Taebi

**Affiliations:** 1 Department of Midwifery, Kashan University of Medical Sciences, Kashan, IR Iran; 2 Trauma Nursing Research Center, Kashan University of Medical Sciences, Kashan, IR Iran; 3 Department of Midwifery, Isfahan University of Medical Sciences, Isfahan, IR Iran

**Keywords:** Nausea, Vomiting, Pregnancy, Ginger

## Abstract

**Background:**

Nausea and vomiting are common and unpleasant complications in pregnancy. Although many alternative therapists support the use of ginger for nausea and vomiting of pregnancy, there is currently insufficient clinical evidence to support its use in this condition

**Objectives:**

The present study was performed to assess the effectiveness of ginger in the treatment of nausea and vomiting in pregnancy.

**Patients and Methods:**

This seven-day clinical trial was performed on 120 eligible pregnant women with symptoms of mild to moderate nausea and vomiting before 16 weeks gestation. They were divided into; ginger, placebo and control groups, by block randomization. Women were asked to record their nausea and vomiting for three days, and then participants received either ginger capsules, or a placebo for four days. No intervention was done with the control group. Data measure was self-recorded symptoms according to the Rhodes Index. Data were analyzed by ANOVA, ANCOVA, Kruskal-Wallis, Chi-square, and Fisher’s exact test, for the quantitative and qualitative variables.

**Results:**

There were no statistical differences in the baseline demographics between the three groups apart from age of marriage and wanted or unwanted pregnancy. An ANCOVA test (covariance test) showed significant differences in mean scores after the intervention in the three groups (P < 0.001).

**Conclusions:**

Ginger was effective for the relief of mild to moderate nausea and vomiting in pregnant women at less than 16 weeks gestation.

## 1. Background

Nausea and vomiting are the most prevalent and probably the most unpleasant complications in pregnancy. It is reported by 50% to 80% of pregnant women (1). There is no known cause for this problem; however, hormonal changes as well as psychological factors may play a role in this condition (2). Approximately, a quarter of all pregnant women have to leave their work because of this problem (3). In less than 2% of cases, this problem escalates to severe nausea and vomiting (hyperemesis gravidarum), and that leads to an imbalance of water and electrolytes, malnutrition, and loss of 5% of body weight (4). This condition may result in the malfunction of different body systems and organs, including the kidneys, and an imbalance of water and electrolytes. In addition, it may also have adverse effects on the fetus (5). Nausea and vomiting in pregnancy (NVP) may lead to depression, feelings of incompetence, loss of work hours, hospitalization and termination of the pregnancy (6). For this reason, an effective treatment during pregnancy is recommended (7). Mothers who experience nausea and vomiting during their pregnancy are more likely to experience the same problem in their next pregnancy (5). Research results have shown that the incidence of NVP in Iran is between 16% to 21.7% (8, 9). 

Antiemetic and non-pharmacological interventions such as acupressure or ginger, are effective in reducing the frequency of nausea and vomiting (2, 10). Ginger is a plant that has been used in traditional medicine for the treatment of all varieties of nausea and vomiting, including NVP (11). The root of the ginger is used to flavor food. In addition, it is used to alleviate gastric discomfort. This root is used in either fresh or dried powder form (12). Some researchers in Iran reported that capsules or biscuits containing ginger are effective in the treatment of NVP (13). The exact mechanism of ginger as an antiemetic agent is not known. It seems that ginger controls serotonin receptors at the level of the digestive system (11). The risks of congenital abnormalities, prenatal mortalities, fetal deaths, low birth weights, and low Apgar scores, do not increase with the use of ginger in pregnancy. Such findings are very important for health care workers who recommend that their pregnant clients use this herbal medicine (14). 

Reports from some studies have shown that ginger can reduce the frequency of NVP (1, 13, 15, 16), but there is no agreement about its dosage (1). While the recommended dose in Europe and North America is no higher than two grams per day, Chinese practitioners prescribe a divided dose of nine grams per day (although they rarely use this amount during pregnancy) (17). The results of a systematic study have mentioned that there are no high-quality studies to support the use of ginger during pregnancy (18). In addition, the results of a study conducted by Lee have demonstrated that the use of ginger during pregnancy does not relieve acute nausea and vomiting (19). Betz et al. in a systematic study, also reported that the use of ginger does not decrease levels of nausea and vomiting following an operation (20). 

## 2. Objectives

Due to the conflicting results in the previous studies and recommendations for further studies in this area, this study was conducted. The aim of our study was to assess the effect of ginger to relieve nausea and vomiting in pregnancy.

## 3. Patients and Methods

This clinical trial was approved by the Ethical Research Committee of Kashan University of Medical Sciences. The participants of this study were pregnant women who were referred to the Prenatal Care Unit of Naghavi Hospital Kashan, Iran. This clinic is the only center that provides prenatal care ten hours per day. The inclusion criterion were:

1. being a volunteer,
2. suffering from nausea and/or mild to moderate vomiting,
3. gestational age less than 16 weeks,
4. singleton pregnancy,
5. reading and writing ability,
6. no digestive disease,
7. no history of treatment with other anti-vomiting drugs within the last three weeks, and
8. residency in Kashan.

The exclusion criterion included: subjects who did not complete the forms, subjects that experienced side effects from consuming ginger capsules, subjects who were advised that the treatment was not effective and that they needed further treatment, and subjects who vomited more than five times per day. 

From December 10, 2008 to July 15, 2009, a total of 120 pregnant women that met the inclusion criterion, were selected from 431 patients referring to the prenatal care center. After obtaining verbal informed consent, a routine pregnancy checkup was completed. Then the participants were divided into three groups (ginger, placebo and control) by using block randomization. All the subjects completed a questionnaire containing demographic information such as; age, age of marriage, gestational age, occupation, parity, wanted or unwanted pregnancy, and education level. They were asked to avoid any kind of medication except for the one prescribed by the researcher. The participants were on this regimen for seven days. They were recommended to increase the number of meals with less volume, reduce high fat and high carbohydrate foods, avoid foods that trigger nausea and vomiting, and to start eating before they felt very hungry. In addition, the participants were asked to avoid smoking, have a piece of dried bread when they woke up, refuse fried, odorous and spicy foods, avoid gas forming drinks, and maintain a suitable position. 

No intervention was made during the first three days and then both placebo and ginger groups received four days treatment. The ginger group received ginger capsules and the placebo group received lactose capsules with a similar shape. They were instructed to seek other treatment if this treatment failed or the frequency of vomiting exceeded five times a day. Every participant received 14 copies of the Rhodes Index form. They also received instructions to complete the form every 12 hours, and to record the intensity of the signs (nausea, vomiting, and retching). This form included eight items that described the signs using a Likert scale ranging from mild (zero) to very severe (four) with a maximum total score of 32. The patient evaluated the syndrome every 12 hours on a 5-point scale (21). This instrument has been used previously in several researches in Iran (8, 22), and in other countries (23, 24). In Iran, its validity was confirmed by content validity method and its reliability was calculated and confirmed by a Cronbach's alpha (α = 0.898) (23). In addition, its reliability was acceptable in other countries (with Cronbach’s alphas of 0.77 in the United Kingdom, 0.897 in the USA, and 0.929 in China) (25-27). 

Every participant in the ginger group received twelve 250 mg ginger capsules produced by Gol Daro Co. (Zintoma trade mark). They were instructed to take three capsules per day, for four days. Similar instructions were given to the placebo group. The researcher contacted every participant twice during the study. Once on the fourth day to answer the women's questions in the three groups and to ask them to start the recommended method. Then again on the seventh day to thank the participants for their participation in the study and to request that they return the Rhodes Index forms for evaluation of their responses to the treatment. NVP was evaluated using the Rhodes Index scores. 

The hypothesis tested was whether ginger resulted in a reduction of nausea, vomiting and retching symptoms. These were indicated by a mean reduction of Rhodes Index scores. 

### 3.1. Ethical Considerations

Eligible women consented to participate in this study. The benefits, risks and effectiveness of the new intervention were described for the women. The privacy of the women and their personal information were protected during the study. The women were asked to start a medication if the advised treatment failed, or vomiting was more than five times per day. The women were informed about the results at the end of the study.

### 3.2. Data Analysis

Results were calculated by the mean Rhodes Index scores in the pre-intervention (three days before the intervention), minus the mean Rhodes Index scores in the post intervention (four days after the intervention), and then compared in the three groups by an ANOVA test. We checked the normal distribution of variables. The ANOVA and Kruskal-Wallis tests were used for normal and non-normal variables respectively. A Chi-square test was performed for the qualitative variables. An ANCOVA test was used to control for confounding variables. A significance level of P < 0.05 was used for all tests. To determine the sample size, a pilot study was conducted on 10 subjects in the ginger and placebo groups, the mean reduction of Rhodes Index scores in the ginger and placebo groups were 7.5 and 3.5 on the Rhodes Index. Thus, the needed sample size with a significance level of 5%, and power of 90%, was calculated to be 33 in each group. Considering a 10% dropout rate, 40 subjects per group were recruited to the study.

## 4. Results

**Figure 1.**
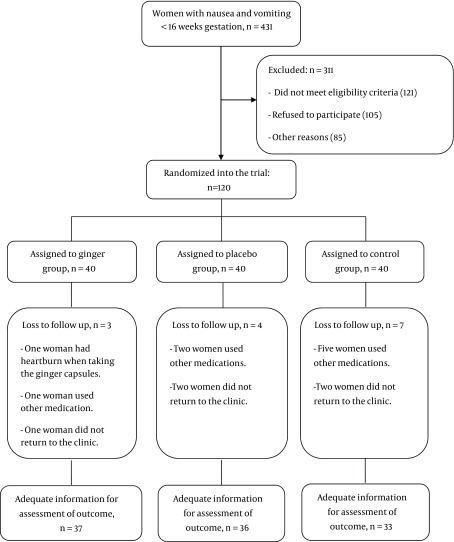
Trial Profile of Recruitment and Randomization to Ginger, Placebo or Control Groups

All of the women were included in the intention to treat (ITT) analysis. During the study, 431 pregnant women were screened, and among them, 120 women were recruited, and 311 were excluded from the study. In the ginger group, one woman had heartburn when she took the ginger capsules, one woman used other medication and one woman did not return to the clinic. In the placebo group, two women used other medications and two women did not return to the clinic. In the control group, five women used other medications and two women did not return to the clinic. These women were excluded from the study. Finally, there were 37 women in the ginger, 36 in the placebo, and 33 in the control groups. Analyses were performed on a total of 106 women (Figure 1). 

There were no statistical differences in the baseline demographics (age, gestational age (weeks), occupation, and level of education) between the three groups apart from age of marriage (P < 0.017), and wanted or unwanted pregnancy (P < 0.027) (Table 1). We compared the mean reduction Rhodes Index scores between the three groups. The resulting scores were significantly greater in the ginger group than in the placebo and control groups. An ANOVA test showed that there were significant differences in the mean reduction of Rhodes Index scores in vomiting, nausea, retching and total scores between the three groups (P < 0.001) (Table 2). A Tukey’s post hoc test was performed and the results showed a significant difference between the three groups in the mean reduction of vomiting, nausea, retching and total Rhodes Index scores (P < 0.001). An ANCOVA test (covariance test) showed that after controlling for the effects of age of marriage and wanted or unwanted pregnancy there were still significant differences in the mean scores of the Rhodes Index after the intervention in the three groups. The percentage reductions for the total Rhodes Index scores were 48% when using ginger, 13% for the placebo, and -10% in the control (Table 3). 

**Table 1.** Baseline Characteristics of the Patients

Characteristics	Ginger, n = 40	Placebo, n = 40	Control, n = 40	P value
**Age, mean ± SD, y**	27.35 ± 5.93	26.85 ± 4.90	25.95 ± 3.46	0.431 ^a^
**Age of marriage mean ± SD, y**	18.9 ± 2.97	20.47 ± 3.34	19.32 ± 1.95	0.02 ^b^
**Gestational age, wk**	8.97 ± 0.05	9.85 ± 2.27	9.30 ± 2.37	0.24 ^a^
**Occupation, No. (%)**				0.578 ^c^
Employee	2 (5)	4 (10)	2 (5)	
Housewife	38 (95)	36 (90)	38 (95)	
**Parity, No. (%)**				0.10 ^d^
Nulliparous	12 (30)	20 (50)	13 (32.5)	
Multiparous	28 (70)	20 (50)	29 (77.5)	
**Pregnancy, No. (%)**				0.027^d^
Wanted	34 (85)	27 (67.5)	36 (90)	
Unwanted	6 (15)	13 (32.5)	4 (10)	
**Level of education, No. (%)**				0.457^d^
Lower than high school	12 (30)	11 (27.5)	16 (40)	
High school and above	28 (70)	29 (72.5)	24 (60)	

^a^ Oneway AVONA was performed.

^b^ Kruskal-Wallis test was performed.

^c^ Fisher’s exact test was performed.

^d^ Chi-square test was performed.

**Table 2.** Mean Reduction of Rhodes Index Scores in the Three Groups

Variable, mean ± SD	Ginger, n = 37	Placebo, n = 36	Control, n = 33	P value ^a^
**Vomiting**	2.52 ± 2.41	0.24 ± 2.24	0.97 ± 2.24	0.001
**Nausea**	3.86 ± 2.35	1.26 ± 1.57	-0.33 ± 1.74	0.001
**Retching**	2.15 ± 1.62	0.45 ± 1.60	-0.34 ± 1.26	0.001
**Total score**	8.53 ± 4.75	1.96 ± 4.02	-1.34 ± 3.88	0.001

^a^ ANOVA test was performed

**Table 3.** The Percentage Reduction of Rhodes Index Scores in the Studied Groups

Variable, %	Ginger, n = 37	Placebo, n = 33	Control, n = 31
**Vomiting**	51	0.7	-36
**Nausea**	46	19	-0.5
**Retching**	26	11	-1
**Total score**	48	13	-10

## 5. Discussion

In this study, we determined the effect of ginger on nausea, vomiting and retching during the first 15 weeks of pregnancy using Rhodes Index forms. There are many studies that have provided information on both pharmacological and non-pharmacological therapies for the management of this condition. Varieties of visual analogue scores, self-reporting tools and Rhodes Index forms (8, 22-24) have been used for the assessment of NVP in previous studies. However, in this study we used Rhodes Index forms. Since the age of marriage and wanted or unwanted pregnancy might influence the results, these variables were controlled by an ANCOVA test. After controlling for these variables, we found a significant reduction in the symptoms of nausea and vomiting in the three groups, although the improvement of nausea and vomiting in the ginger group was statistically greater than in the placebo group. 

Ozgoli found that ginger was an effective herbal remedy for decreasing nausea and vomiting, and most of the women in the ginger group reported an improvement in their nausea symptoms during pregnancy (13). This study confirms the presence of a placebo effect in the relief of nausea, but it still detected some benefits of ginger in improving NVP. In Willetts’ study, the nausea experience score was significantly less for the ginger extract group compared to the placebo group after the first day of treatment, and this difference was present on each treatment day. Retching was also reduced by the ginger extract, although to a lesser extent. No significant effect was observed on vomiting (28). The reason might be due to the dose of ginger used in Willetts’ study, which was 500 mg compare to the 750 mg that was used in our study. The results in the present study showed that vomiting and nausea in the ginger group decreased by 51% and 46%, respectively. Total percentage reduction of Rhodes Index scores in the present study were 48%, 13%, and 10%, in the ginger, placebo, and control groups, respectively. These findings indicate that ginger is more effective in treating nausea and vomiting than a placebo. 

In conclusion, ginger is effective in reducing NVP in mild to moderate symptomatic pregnant women before 16 weeks gestation. Since this study was performed on mild to moderate nausea and vomiting, the results are not generalized to severe nausea and vomiting. The effectiveness of ginger and other non-pharmacological therapies such as acupressure in relieving severe NVP could be investigated in future clinical trials. 
